# *N*-(5-Amino-1*H*-1,2,4-triazol-3-yl)pyridine-2-carboxamide

**DOI:** 10.1107/S1600536813000123

**Published:** 2013-01-12

**Authors:** Javier Hernández-Gil, Sacramento Ferrer, Rafael Ballesteros, Alfonso Castiñeiras

**Affiliations:** a Departament de Química Inorgànica, Universitat de València, Vicent Andrés Estellés s/n, 46100 Burjassot, València, Spain; b Departament de Química Orgànica, Universitat de València, Vicent Andrés Estellés s/n, 46100 Burjassot, València, Spain; c Deptament de Química Inorgánica, Universitat de Santiago de Compostela, Campus vida, Santiago de Compostela, Galicia, 15782, Spain

## Abstract

The title compound, C_8_H_8_N_6_O, was obtained by the reaction of 3,5-diamino-1,2,4-triazole with ethyl 2-picolinate in a glass oven. The dihedral angles formed between the plane of the amide group and the pyridine and triazole rings are 11.8 (3) and 5.8 (3)°, respectively. In the crystal, an extensive system of classical N—H⋯N and N—H⋯O hydrogen bonds generate an infinite three-dimensional network.

## Related literature

For background to triazole derivatives, see: Aromí *et al.* (2011 ▶); Olguín *et al.* (2012 ▶). For related triazole structures, see: Allouch *et al.* (2008 ▶); Ouakkaf *et al.* (2011 ▶). For structures of metal complexes with related triazoles, see: Ferrer *et al.* (2004 ▶, 2012 ▶). For the synthesis of triazoles, see: Chernyshev *et al.* (2005 ▶). For hydrogen-bond motifs, see: Bernstein *et al.* (1995 ▶).
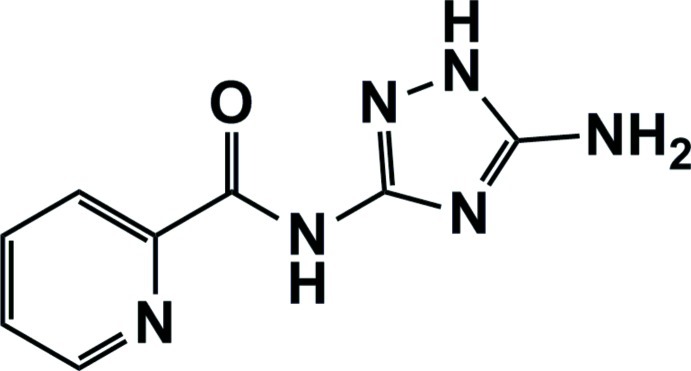


## Experimental

### 

#### Crystal data

C_8_H_8_N_6_O
*M* *_r_* = 204.20
Tetragonal, 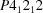

*a* = 9.5480 (5) Å
*c* = 21.9570 (9) Å
*V* = 2001.69 (17) Å^3^
*Z* = 8
Mo *K*α radiation
μ = 0.10 mm^−1^
*T* = 293 K
0.15 × 0.09 × 0.05 mm

#### Data collection

Nonius KappaCCD diffractometer
4484 measured reflections
1407 independent reflections
915 reflections with *I* > 2σ(*I*)
*R*_int_ = 0.048

#### Refinement

*R*[*F* ^2^ > 2σ(*F* ^2^)] = 0.038
*wR*(*F* ^2^) = 0.107
*S* = 1.07
1407 reflections
137 parameters
H-atom parameters constrained
Δρ_max_ = 0.12 e Å^−3^
Δρ_min_ = −0.13 e Å^−3^

### 

Data collection: *COLLECT* (Nonius, 1998 ▶); cell refinement: *DENZO* and *SCALEPACK* (Otwinowski & Minor, 1997 ▶); data reduction: *DENZO* and *SCALEPACK*; program(s) used to solve structure: *SHELXS97* (Sheldrick, 2008 ▶); program(s) used to refine structure: *SHELXL97* (Sheldrick, 2008 ▶); molecular graphics: *PLATON* (Spek, 2009 ▶) and *DIAMOND* (Brandenburg & Putz, 2006 ▶); software used to prepare material for publication: *publCIF* (Westrip, 2010 ▶).

## Supplementary Material

Crystal structure: contains datablock(s) I, global. DOI: 10.1107/S1600536813000123/gk2547sup1.cif

Structure factors: contains datablock(s) I. DOI: 10.1107/S1600536813000123/gk2547Isup2.hkl

Supplementary material file. DOI: 10.1107/S1600536813000123/gk2547Isup3.cml

Additional supplementary materials:  crystallographic information; 3D view; checkCIF report

## Figures and Tables

**Table 1:** Hydrogen-bond geometry (Å, °)

*D*—H⋯*A*	*D*—H	H⋯*A*	*D*⋯*A*	*D*—H⋯*A*
N21—H21⋯N23^i^	0.86	2.02	2.788 (3)	149
N21—H21⋯O17^i^	0.86	2.41	3.061 (3)	133
N18—H18⋯N20^ii^	0.86	2.45	3.253 (3)	155
N22—H22*A*⋯O17^i^	0.86	2.08	2.860 (3)	150
N22—H22*B*⋯N20^iii^	0.86	2.26	3.068 (3)	157

Symmetry codes: (i) 
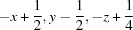
; (ii) 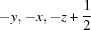; (iii) 
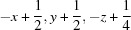
.

**Fig. 1.**
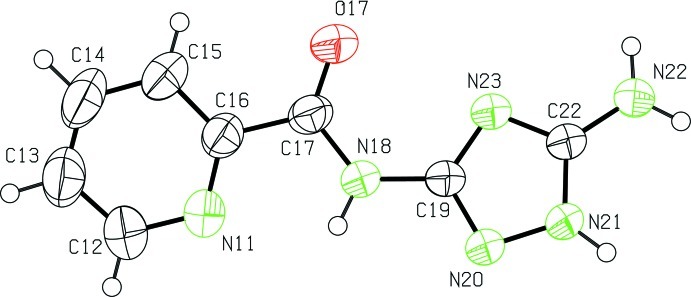
Molecular structure of the title molecule with atom-labelling scheme. Displacement ellipsoids are drawn at the 50% probability level and H atoms are shown as spheres of arbitrary radii.

**Fig. 2.**
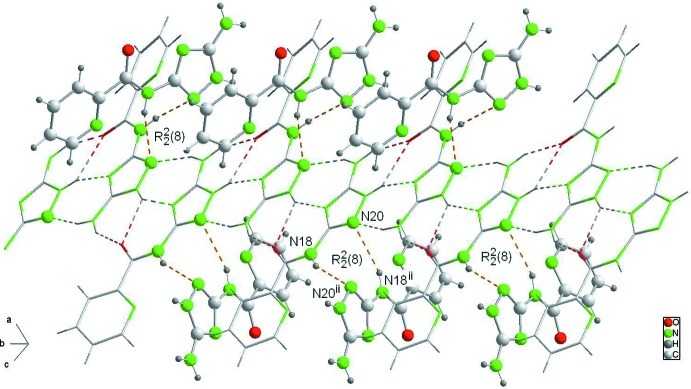
Scheme with details of the crossing of two chains of molecules (along the cb plane). Hydrogen bonds are shown as dashed lines. Symmetry code: (ii) -y, -x, -z + 1/2

**Fig. 3.**
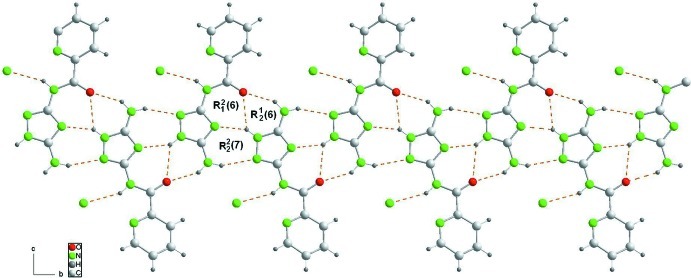
Tapes of title molecules via N—H···N and N—H···O interactions seen along the [100] direction. Hydrogen bonds are shown as orange dashed lines.

**Fig. 4.**
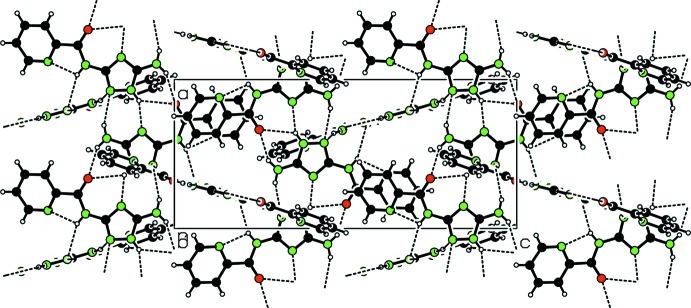
A view of the unit-cell content of the title compound in projection down the b axis. Hydrogen bonds are shown as dashed lines.

## supplementary crystallographic information

### Comment

A significantly large variety of 1,2,4-triazole-based compounds have been prepared to serve as ligands with the aim of obtaining discrete polynuclear metal complexes or polymeric coordination networks, owing to the ability of the 1,2,4-triazole ring to bridge metal ions through different coordination ways (Aromí *et al.*, 2011; Olguín *et al.*, 2012). Usually the 1,2,4-triazole-based family of ligands are classified in three categories (Aromí *et al.*, 2011): (*a*) those containing an unique coordinative ring, (*b*) those possessing two or more coordinative rings linked by a spacer, and (*c*) the *mixed* ligands, which present two or more functional groups. Most of the 3,5-disubstituted derivatives can be included in the last category (Allouch *et al.*, 2008; Ouakkaf *et al.*, 2011).

Our group has been reporting on the synthesis and structure of some 3,5-disubstituted triazole-based ligands, *i.e.* 5-amino-3-pyridin-2-yl-1,2,4-triazole (Ferrer *et al.*, 2004) and 3-acetylamino-5-amino-1,2,4-triazole (Ferrer *et al.*, 2012). In those cases, single crystals of the ligands suitable for X-ray analysis were not obtained. Instead, crystal structures of some of their Cu^II^ complexes could be determined, thus confirming the structure of the triazole, either in neutral or in anionic form. In this work we describe a novel compound of this series, namely: 5-amino-3-picolinamido-1*H*-1,2,4-triazole or 5-amino-3-(pyridin-2-yl-acetamido)-1*H*-1,2,4-triazole (abbreviated as H_2_V to account for the presence of two acidic H atoms), for which it has been possible to solve the crystal structure.

The obtained H_2_V species is an attractive ligand since it presents 5 to 7 donor atoms (depending on the degree of deprotonation) but also the possibility of forming different chelating rings when coordinated to metals. Besides, in metal complexes the pyridyl ring often rotates around the single C–C bond leading to different binding conformations (Ouakkaf *et al.*, 2011). This enlarges its capability to produce novel metal-organic structures.

As shown in Figure 1, the NH hydrogen is *trans* to the C=O group, as is observed for all N monosustituted amides. Molecular dimensions, such as the C=O bond length of 1.227 (3) Å and the central C–N–C amide angle of 127.40 (17)°, may be considered normal.

In the crystal packing, the triazole ligands are linked by pairs of weak N—H···N hydrogen bonds involving the H18 and N20 atoms, thus generating a characteristic *R*^2^_2_(8) ring motif (Bernstein *et al.*, 1995) (Fig. 2). Moreover, the molecules are also linked by N—H···N and N—H···O hydrogen bonds, forming fused non-centrosymmetric rings *R*^2^_2_(7), *R*^2^_1_(6) and *R*^1^_2_(6) and giving rise to one-dimensional tapes parallel to the [010] and [100] directions (Fig. 3). These tapes joined by the R_2_^2^(8) motif of N-H···N hydrogen bonds form a three dimensional framework (Fig.4).

### Experimental

An evaporating flask containing 3,5-diamino-1,2,4-triazole (41.4 mmol, 4.10 g) and ethyl 2-picolinate (6.3 ml, 7 g, 46.3 mmol) was connected to a glass oven and the reaction temperature was slowly raised to 210 °C. The mixture was stirred (rotated) for 4 h. At this point, a vacuum pump was connected during 60 minutes to remove the excess of ethyl 2-picolinate. Afterwards, the reaction was cooled down to room temperature and the mixture solidified. The crude product was washed with ethanol and acetone and then recrystallized from methanol to give analytically pure crystals.

### Refinement

All H atoms were positioned geometrically and were treated as riding on their parent atoms, with C—H distances of 0.93 Å and N—H distances of 0.86 Å with *U*_iso_(H) = 1.2*U*_eq_(C/N). In the absence of significant anomalous dispersion, Friedel pairs were merged.

### Crystal data

**Table:**

C_8_H_8_N_6_O	*D*_x_ = 1.355 Mg m^−^^3^
*M**_r_* = 204.20	Melting point: 494(1) K
Tetragonal, *P*4_1_2_1_2	Mo *K*α radiation, λ = 0.71073 Å
Hall symbol: P 4abw 2nw	Cell parameters from 2353 reflections
*a* = 9.5480 (5) Å	θ = 1.0–27.5°
*c* = 21.9570 (9) Å	µ = 0.10 mm^−^^1^
*V* = 2001.69 (17) Å^3^	*T* = 293 K
*Z* = 8	Prism, colourless
*F*(000) = 848	0.15 × 0.09 × 0.05 mm

### Data collection

**Table:**

Nonius KappaCCD diffractometer	915 reflections with *I* > 2σ(*I*)
Radiation source: fine-focus sealed tube	*R*_int_ = 0.048
Graphite monochromator	θ_max_ = 27.5°, θ_min_ = 2.3°
Detector resolution: 9 pixels mm^-1^	*h* = −12→12
ω and phi scans	*k* = −8→8
4484 measured reflections	*l* = −28→27
1407 independent reflections	

### Refinement

**Table:**

Refinement on *F*^2^	Secondary atom site location: difference Fourier map
Least-squares matrix: full	Hydrogen site location: difference Fourier map
*R*[*F*^2^ > 2σ(*F*^2^)] = 0.038	H-atom parameters constrained
*wR*(*F*^2^) = 0.107	*w* = 1/[σ^2^(*F*_o_^2^) + (0.0557*P*)^2^ + 0.0085*P*] where *P* = (*F*_o_^2^ + 2*F*_c_^2^)/3
*S* = 1.07	(Δ/σ)_max_ < 0.001
1407 reflections	Δρ_max_ = 0.12 e Å^−^^3^
137 parameters	Δρ_min_ = −0.13 e Å^−^^3^
0 restraints	Extinction correction: *SHELXL97* (Sheldrick, 2008), Fc^*^=kFc[1+0.001xFc^2^λ^3^/sin(2θ)]^-1/4^
Primary atom site location: structure-invariant direct methods	Extinction coefficient: 0.013 (2)

### Special details

**Table:**

Geometry. All e.s.d.'s (except the e.s.d. in the dihedral angle between two l.s. planes) are estimated using the full covariance matrix. The cell e.s.d.'s are taken into account individually in the estimation of e.s.d.'s in distances, angles and torsion angles; correlations between e.s.d.'s in cell parameters are only used when they are defined by crystal symmetry. An approximate (isotropic) treatment of cell e.s.d.'s is used for estimating e.s.d.'s involving l.s. planes.
Refinement. Refinement of *F*^2^ against ALL reflections. The weighted *R*-factor *wR* and goodness of fit *S* are based on *F*^2^, conventional *R*-factors *R* are based on *F*, with *F* set to zero for negative *F*^2^. The threshold expression of *F*^2^ > σ(*F*^2^) is used only for calculating *R*-factors(gt) *etc*. and is not relevant to the choice of reflections for refinement. *R*-factors based on *F*^2^ are statistically about twice as large as those based on *F*, and *R*- factors based on ALL data will be even larger.

### Fractional atomic coordinates and isotropic or equivalent isotropic displacement parameters (Å^2^)

**Table:**

	*x*	*y*	*z*	*U*_iso_*/*U*_eq_	
O17	0.1793 (2)	0.33529 (18)	0.25244 (8)	0.0726 (6)	
N11	0.0462 (3)	0.1210 (3)	0.36912 (9)	0.0708 (7)	
N18	0.1596 (2)	0.09855 (19)	0.25946 (8)	0.0513 (5)	
H18	0.1399	0.0318	0.2843	0.062*	
N20	0.2076 (2)	−0.0746 (2)	0.18712 (8)	0.0495 (5)	
N21	0.2450 (2)	−0.0695 (2)	0.12653 (8)	0.0503 (5)	
H21	0.2593	−0.1412	0.1036	0.060*	
N22	0.2887 (3)	0.1049 (2)	0.05253 (9)	0.0797 (9)	
H22A	0.3046	0.0438	0.0246	0.096*	
H22B	0.2939	0.1928	0.0442	0.096*	
N23	0.2279 (2)	0.14920 (19)	0.15549 (8)	0.0543 (6)	
C12	−0.0006 (4)	0.1273 (4)	0.42633 (13)	0.0885 (11)	
H12	−0.0387	0.0467	0.4434	0.106*	
C13	0.0043 (4)	0.2465 (4)	0.46156 (14)	0.0871 (10)	
H13	−0.0288	0.2460	0.5014	0.104*	
C14	0.0583 (5)	0.3637 (4)	0.43690 (15)	0.1011 (12)	
H14	0.0627	0.4458	0.4596	0.121*	
C15	0.1072 (4)	0.3613 (3)	0.37768 (13)	0.0877 (11)	
H15	0.1451	0.4412	0.3599	0.105*	
C16	0.0983 (3)	0.2377 (3)	0.34567 (11)	0.0585 (7)	
C17	0.1488 (3)	0.2302 (3)	0.28160 (11)	0.0541 (6)	
C19	0.1992 (2)	0.0590 (2)	0.20093 (10)	0.0463 (6)	
C22	0.2557 (3)	0.0633 (2)	0.10904 (10)	0.0503 (6)	

### Atomic displacement parameters (Å^2^)

**Table:**

	*U*^11^	*U*^22^	*U*^33^	*U*^12^	*U*^13^	*U*^23^
O17	0.1132 (17)	0.0408 (10)	0.0639 (11)	−0.0008 (10)	0.0054 (11)	0.0032 (9)
N11	0.0937 (19)	0.0633 (16)	0.0552 (13)	−0.0053 (13)	0.0148 (12)	−0.0079 (11)
N18	0.0701 (14)	0.0370 (11)	0.0468 (11)	−0.0015 (10)	0.0061 (10)	−0.0016 (9)
N20	0.0677 (14)	0.0356 (11)	0.0453 (10)	0.0003 (9)	0.0062 (9)	−0.0003 (9)
N21	0.0723 (14)	0.0345 (11)	0.0442 (10)	0.0013 (10)	0.0061 (9)	−0.0020 (8)
N22	0.147 (3)	0.0395 (13)	0.0522 (13)	0.0045 (14)	0.0264 (15)	0.0046 (10)
N23	0.0790 (15)	0.0351 (10)	0.0487 (11)	−0.0009 (10)	0.0068 (11)	0.0014 (9)
C12	0.115 (3)	0.085 (2)	0.0647 (18)	−0.010 (2)	0.0275 (18)	−0.0075 (17)
C13	0.104 (3)	0.093 (3)	0.0643 (18)	0.014 (2)	0.0149 (17)	−0.0163 (19)
C14	0.148 (4)	0.080 (3)	0.076 (2)	0.018 (3)	0.009 (2)	−0.033 (2)
C15	0.136 (3)	0.0541 (19)	0.073 (2)	0.0064 (19)	0.0134 (19)	−0.0150 (16)
C16	0.0715 (18)	0.0512 (17)	0.0528 (14)	0.0080 (13)	0.0001 (12)	−0.0059 (13)
C17	0.0664 (16)	0.0402 (14)	0.0558 (14)	0.0025 (12)	−0.0052 (12)	−0.0005 (12)
C19	0.0576 (15)	0.0364 (13)	0.0448 (13)	−0.0009 (11)	0.0011 (11)	0.0022 (10)
C22	0.0686 (17)	0.0355 (13)	0.0468 (13)	−0.0007 (12)	0.0043 (12)	−0.0005 (11)

### Geometric parameters (Å, º)

**Table:**

O17—C17	1.225 (3)	N22—H22B	0.8600
N11—C16	1.324 (3)	N23—C22	1.335 (3)
N11—C12	1.335 (3)	N23—C19	1.347 (3)
N18—C17	1.352 (3)	C12—C13	1.377 (4)
N18—C19	1.392 (3)	C12—H12	0.9300
N18—H18	0.8600	C13—C14	1.346 (5)
N20—C19	1.314 (3)	C13—H13	0.9300
N20—N21	1.378 (2)	C14—C15	1.382 (4)
N21—C22	1.329 (3)	C14—H14	0.9300
N21—H21	0.8600	C15—C16	1.376 (4)
N22—C22	1.340 (3)	C15—H15	0.9300
N22—H22A	0.8600	C16—C17	1.489 (3)
			
C16—N11—C12	117.0 (2)	C13—C14—C15	119.6 (3)
C17—N18—C19	127.3 (2)	C13—C14—H14	120.2
C17—N18—H18	116.4	C15—C14—H14	120.2
C19—N18—H18	116.3	C16—C15—C14	118.3 (3)
C19—N20—N21	101.78 (18)	C16—C15—H15	120.9
C22—N21—N20	109.41 (18)	C14—C15—H15	120.9
C22—N21—H21	125.3	N11—C16—C15	123.1 (2)
N20—N21—H21	125.3	N11—C16—C17	116.7 (2)
C22—N22—H22A	120.0	C15—C16—C17	120.2 (3)
C22—N22—H22B	120.0	O17—C17—N18	123.7 (2)
H22A—N22—H22B	120.0	O17—C17—C16	122.1 (2)
C22—N23—C19	102.32 (19)	N18—C17—C16	114.1 (2)
N11—C12—C13	123.7 (3)	N20—C19—N23	116.0 (2)
N11—C12—H12	118.1	N20—C19—N18	119.6 (2)
C13—C12—H12	118.1	N23—C19—N18	124.4 (2)
C14—C13—C12	118.3 (3)	N21—C22—N23	110.5 (2)
C14—C13—H13	120.8	N21—C22—N22	124.6 (2)
C12—C13—H13	120.8	N23—C22—N22	124.9 (2)
			
C19—N20—N21—C22	−0.1 (3)	N11—C16—C17—N18	−12.1 (4)
C16—N11—C12—C13	−0.8 (6)	C15—C16—C17—N18	167.8 (3)
N11—C12—C13—C14	0.6 (6)	N21—N20—C19—N23	−0.2 (3)
C12—C13—C14—C15	−0.2 (6)	N21—N20—C19—N18	178.5 (2)
C13—C14—C15—C16	0.2 (5)	C22—N23—C19—N20	0.5 (3)
C12—N11—C16—C15	0.8 (5)	C22—N23—C19—N18	−178.2 (2)
C12—N11—C16—C17	−179.3 (3)	C17—N18—C19—N20	178.4 (2)
C14—C15—C16—N11	−0.5 (5)	C17—N18—C19—N23	−3.0 (4)
C14—C15—C16—C17	179.6 (3)	N20—N21—C22—N23	0.4 (3)
C19—N18—C17—O17	−3.3 (4)	N20—N21—C22—N22	−179.1 (3)
C19—N18—C17—C16	177.7 (2)	C19—N23—C22—N21	−0.5 (3)
N11—C16—C17—O17	168.9 (3)	C19—N23—C22—N22	179.0 (3)
C15—C16—C17—O17	−11.3 (4)		

### Hydrogen-bond geometry (Å, º)

**Table:**

*D*—H···*A*	*D*—H	H···*A*	*D*···*A*	*D*—H···*A*
N21—H21···N23^i^	0.86	2.02	2.788 (3)	149
N21—H21···O17^i^	0.86	2.41	3.061 (3)	133
N18—H18···N20^ii^	0.86	2.45	3.253 (3)	155
N22—H22*A*···O17^i^	0.86	2.08	2.860 (3)	150
N22—H22*B*···N20^iii^	0.86	2.26	3.068 (3)	157

Symmetry codes: (i) −*x*+1/2, *y*−1/2, −*z*+1/4; (ii) −*y*, −*x*, −*z*+1/2; (iii) −*x*+1/2, *y*+1/2, −*z*+1/4.
